# AMP-Activated Protein Kinase as a Key Trigger for the Disuse-Induced Skeletal Muscle Remodeling

**DOI:** 10.3390/ijms19113558

**Published:** 2018-11-12

**Authors:** Natalia A. Vilchinskaya, Igor I. Krivoi, Boris S. Shenkman

**Affiliations:** 1 Myology Laboratory, Institute of Biomedical Problems RAS, Moscow 123007, Russia; vilchinskayanatalia@gmail.com; 2 Department of General Physiology, St. Petersburg State University, St. Petersburg 199034, Russia; iikrivoi@gmail.com

**Keywords:** AMPK, HDAC4/5, p70S6K, MyHC I(β), motor endplate remodeling, soleus muscle, mechanical unloading, hindlimb suspension

## Abstract

Molecular mechanisms that trigger disuse-induced postural muscle atrophy as well as myosin phenotype transformations are poorly studied. This review will summarize the impact of 5′ adenosine monophosphate -activated protein kinase (AMPK) activity on mammalian target of rapamycin complex 1 (mTORC1)-signaling, nuclear-cytoplasmic traffic of class IIa histone deacetylases (HDAC), and myosin heavy chain gene expression in mammalian postural muscles (mainly, soleus muscle) under disuse conditions, i.e., withdrawal of weight-bearing from ankle extensors. Based on the current literature and the authors’ own experimental data, the present review points out that AMPK plays a key role in the regulation of signaling pathways that determine metabolic, structural, and functional alternations in skeletal muscle fibers under disuse.

## 1. Introduction

Skeletal muscle is a highly plastic organ, which is able to change its structure and metabolism depending on the mode of contractile activity. Such conditions as hypokinesia, immobilization, paralysis, and weightlessness can lead to a complex of atrophic changes (most pronounced in postural muscles), resulting from a significant reduction in muscle mass and contractile function [1,2]. Skeletal muscle disuse also leads to a reduction in muscle stiffness and slow-to-fast myosin phenotype transformations [2,3,4,5,6,7]. Muscle atrophy observed during muscle inactivation under conditions of real and simulated microgravity, joint immobilization, or spinal isolation is associated with an increase in proteolytic processes and a decrease in protein synthesis [4,8,9,10,11]. Myosin phenotype shift occurs as a result of a decrease in the gene expression of the slow isoform of myosin heavy chain (MyHC) and an increase in the expression of the fast MyHC isoforms [12,13,14].

To study the mechanisms of muscle disuse atrophy, a variety of experimental models with the different rate of reduction in muscle electrical and contractile activity are used. In this sense, one of the most suitable models is a rodent hindlimb suspension (HS) technique, which prevents the hindlimbs from touching any supporting surface, resulting in a cessation of rat soleus neuromuscular activity [15,16,17]. Similar effects are observed during dry immersion in human skeletal muscle [1,18,19]. These models not only provide an almost complete cessation of the soleus muscle contractile activity, simulating the effects of weightlessness, but also allow the experimentalist to avoid invasive procedures (denervation, spinal isolation, administration of toxins, etc.). Hence, this review will mainly summarize the data obtained in HS and dry immersion models.

Despite a large number of studies aimed at the analysis of disuse muscle atrophy, the triggering mechanisms of its development within a few hours/days after withdrawal of weight-bearing from postural muscles are still poorly studied [1,15,16]. The earliest effects of unloading (the first 24 h) on postural muscle include: (1) Depolarization of the sarcolemma due to an inactivation of the α2 subunit of the Na,K-ATPase [20,21], (2) disintegration of cholesterol rafts [22], and (3) translocation of the neuronal NO-synthase from the subsarcolemmal compartment to the cytoplasm [23]. However, the mechanisms of development of these changes and, most importantly, the dependence of these mechanisms on molecular triggers determined by the level of muscle contractile activity/inactivity remain unknown.

It seems natural that the reduction/cessation of electrical and, accordingly, contractile activity of the muscle can lead to changes in the basic physiological mechanisms that directly depend on the activity of muscle fibers.

1. Changes in electrogenic signaling mechanisms due to termination of electrical activity (hypothetical decrease in Na^+^ concentration inside the muscle fibers, the expected temporary cessation of Ca^2+^ flow through voltage-dependent L-type calcium channels).

2. Mechanosensory molecular changes due to termination of the mechanical action of extracellular matrix structures on mechanosensory molecules (changes in the state of integrins, etc. [24], termination of the active state of actin stress-fibers, inactivation of mechanosensitive channels, inactivation of mechanosensory myofibrillar proteins).

3. Changes in energy metabolism as a result of termination of ATP expenditure (changes in the ratio of ATP/ADP/AMP and PCr/Cr, accumulation of glycogen and reactive oxygen species).

It is not yet possible to trace the entire complex of processes that link the cessation of electrical activity and the elimination of mechanical loading of muscle fibers with the development of early molecular events during mechanical unloading. As for the consequences of the cessation of metabolic energy expenditure during unloading, the situation is somewhat different. In the 1990s, it was hypothesized that mechanical unloading leads to a change in the balance of high-energy phosphate compounds towards the accumulation of fully phosphorylated compounds [25]. Wakatsuki and co-authors have shown the accumulation of phosphocreatine in the soleus muscle of rats after 10 days of HS [26]. In 1987, Henriksen and Tischler reported a 25% increase in glycogen content in rat soleus during the first three days of HS [27].

**Figure 1:**
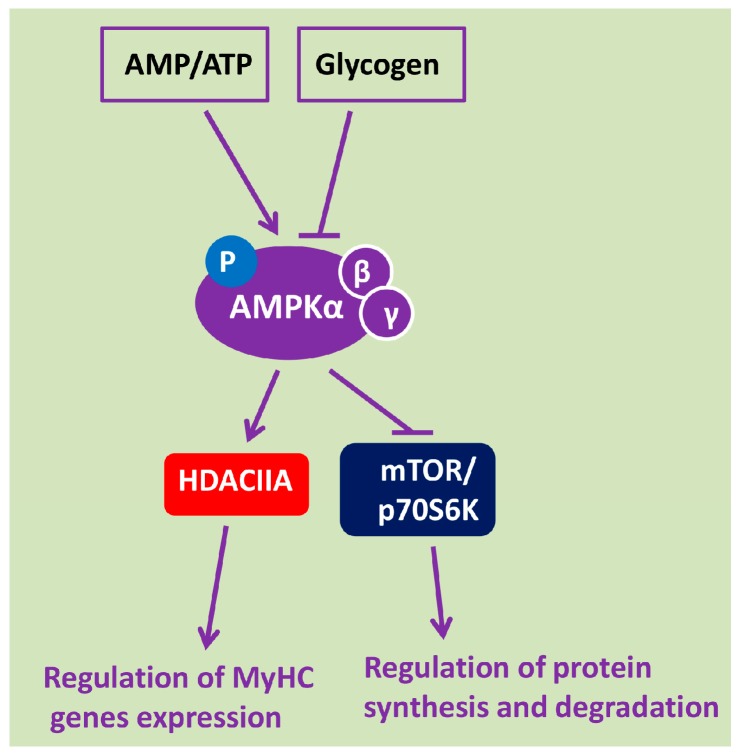
Key physiological regulators of 5′ adenosine monophosphate -activated protein kinase (AMPK) activity in skeletal muscle and physiologically-relevant AMPK targets. (original scheme)

If the described changes in energy metabolism are the potential triggers for signaling processes leading to the development of postural muscle atrophy, reduced intrinsic muscle stiffness, and myosin phenotype shift, there should be a specific sensor of the state of energy metabolism. Such a sensor has long been known. It is 5′ adenosine monophosphate -activated protein kinase (AMPK), the cell’s main energy sensor reacting to the changes in the ratio of high-energy phosphates (Figure 1). Therefore, the termination of electrical activity in soleus muscle at the initial stage of gravitational unloading should affect AMPK activity.

## 2. AMPK Is a Key Energy Sensor and Metabolic Regulator of Signaling Pathways in Skeletal Muscle Fibers

AMPK is involved in transmission of extracellular and intracellular signals by phosphorylation of various substrates in many metabolic reactions in skeletal muscle. AMRK is a heterotrimeric complex consisting of three proteins: α-subunit, which has its own kinase activity, and two regulatory subunits, β and γ [28].

AMPK activity is regulated both allosterically and by post-translational modifications (phosphorylation). Allosteric activation of AMPK is carried out with the help of AMP and its analogues. Phosphorylation AMPK at Thr172 of the α-subunit leads to its activation. This phosphorylation is regulated by calcium-/calmodulin-dependent kinase kinase 2 (CaMKK2) and liver kinase B1 (LKB1). Further activation of AMPK occurs due to conformational changes occurring upon binding of AMP or ADP to the γ-subunit of AMPK, promoting Thr172 phosphorylation at the α-subunit. The combined effect of Thr172 phosphorylation of the α-subunit of AMPK and allosteric regulation leads to more than a 1000-fold increase in AMPK activity, which makes AMPK highly sensitive to alternations in the energy status of the cell [29]. 

Activation of AMPK may also occur under the action of extracellular regulatory factors, such as interleukin-6 (IL-6) and brain-derived neurotrophic factor (BDNF) [30]. Interestingly, AMPK is involved in the regulation of IL-6 expression in skeletal muscles [31]. One of the factors of AMPK activation is nitric oxide (NO), the production of which is determined by the activity of neuronal and endothelial NO synthases [32,33]. There is evidence that AMPK activation can result from mechanical stretch via components of the dystrophin-glycoprotein complex (at least in cardiomyocytes) [34].

AMPK can also be phosphorylated on Ser485/491 sites by protein kinase D and some isoforms of protein kinase C [35], which leads to inhibition of AMPK activity. A decrease in AMPK activity is associated with increased glycogen content, as well as accumulation of ATP and creatine phosphate [36,37]. AMPK has a number of molecular targets in skeletal muscle. It is known that AMPK can activate Na,K-ATPase [38,39] and phosphorylate neuronal NO-synthase [40]. AMPK is also involved in the regulation of protein synthesis and degradation [36]. AMPK is a negative regulator of protein synthesis in skeletal muscle. This kinase can inhibit the key regulator of protein synthesis, the mammalian target of rapamycin complex 1 (mTORC1), through phosphorylation of TSC2 [41] and raptor [42]. AMPK can also be involved in the degradation of myofibrillar proteins [43]. Nakashima and co-authors have shown that AMPK participates in the degradation of myofibrillar proteins through the activation of forkhead box proteins (FOXO) transcription factors and subsequent up-regulation of muscle-specific E3 ubiquitin-ligases atrogin-1/MAFbx and MuRF-1 [44].

In addition, AMPK, as a key energy sensor of the cell regulating energy metabolism, participates in the initiation of autophagy [45]. AMPK can directly phosphorylate Unc-51-like kinase (ULK-1) across several sites as well as activate autophagy by inhibiting mTOR activity [46,47].

In recent years, it has been shown that AMPK can influence the expression of a number of genes by phosphorylation of class IIA histone deacetylases (HDAC4, HDAC5, HDAC7), leading to their exclusion from the nucleus and activation of gene expression [48,49,50]. 

Thus, according to modern concepts, AMPK activity is mainly determined by the state of energy metabolism: AMPK activity increases with increased ATP consumption, AMP accumulation, and glycogen depletion, and decreases with the accumulation of ATP and glycogen in muscle fibers. Activated AMPK phosphorylates and retains class IIA HDACs outside the myonuclei (thereby contributing to the expression of a number of genes) and inhibits the activity of mTORC1 and its primary targets (Figure 1). Dephosphorylated AMPK, on the contrary, promotes HDACs nuclear import and transcriptional suppression of gene expression, while reducing the degree of mTORC1 suppression.

## 3. AMPK Activity under Conditions of Mechanical Unloading

Until recently, AMPK activity/phosphorylation in skeletal muscle under unloading conditions has been poorly studied. Moreover, the literature reveals contradictory results concerning AMPK Thr172 phosphorylation at various time-points in different models of disuse. It has been shown that four- and seven-day denervation resulted in a significant increase in AMPK phosphorylation in rodent skeletal muscles [51,52], while deletion of AMPKα2 significantly attenuated denervation-induced skeletal muscle wasting and protein degradation [51]. In human skeletal muscle with recent complete cervical spinal cord injury, AMPKα2 protein abundance decreased by 25% during the first year after injury, without significant change in AMPKα1 content. Furthermore, AMPK phosphorylation on Thr172 was significantly decreased during the first year post-spinal cord injury in human vastus lateralis muscle [53]. Thirty-day space flight and subsequent recovery did not affect AMPK Thr172 phosphorylation in murine longissimus dorsi muscle [54]. In terms of fiber-type composition, murine longissimus dorsi is similar to soleus muscle. However, it should be noted that it is not quite correct to compare the data obtained from rat and mouse soleus muscle. It is known that rat soleus muscle, as well as human soleus, comprises about 85% of slow-twitch fibers expressing the slow isoform of MyHC, while mouse soleus consists of approximately 40% slow-twitch fibers [55]. Obviously, this fact determines the essential features of metabolism in the mouse soleus muscle and its response to gravitational unloading. Therefore, unloading-induced changes in mouse soleus can significantly differ from that of rats and humans. 

Vilchinskaya et al. (2015) have shown for the first time that short-term (three days) gravitational unloading via dry immersion leads to a significant decrease in the level of AMPK Thr172 phosphorylation in human soleus muscle [56]. The literature data on the AMPK activity in rat soleus following HS are inconsistent. AMPK activity, which is usually assessed by the level of Thr172 phosphorylation [57,58,59], was reported to be reduced in rat soleus after two weeks of HS [60], whereas Hilder and co-authors showed that 14-day HS results in a significant increase in AMPK Thr172 phosphorylation in rat soleus [61]. At the same time, Egawa and others did not find any changes in AMPKα1 и AMPKα2 activity in mouse soleus after 14-day HS, however, the level of ACC phosphorylation was upregulated [62,63]. 

A significant reduction in AMPK Thr172 phosphorylation was previously observed in rat soleus at the early stage (24 h) of hindlimb unloading [11]. A recent study has also demonstrated a significant decrease in AMPK Thr172 phosphorylation in rat soleus muscle already after 6- and 12-h mechanical unloading [64]. In an inactive skeletal muscle, a rapid accumulation of completely phosphorylated high-energy phosphates can occur, resulting in reduced AMPK activity. Thus, AMPK dephosphorylation at the early stage of gravitational unloading can be caused by a decrease in postural muscle energy consumption and a corresponding change in the ratio of phosphorylated and dephosphorylated adenine nucleotides (ATP, ADP, AMP). 

It is known that binding of AMPK to glycogen results in reduced AMPK activity [37]. Therefore, it is possible that a decrease in the activity of AMPK at the initial stages of mechanical unloading may be associated with glycogen accumulation. Indeed, glycogen concentration in rat soleus muscle during the first three days of HS is significantly increased [27]. 

After seven-day HS, AMPK phosphorylation does not differ from that of control [11], which correlates well with the restoration of electromyographic activity of rat soleus following six to seven days of HS [15]. Moreover, 14-day HS resulted in a significant increase in AMPK Thr172 phosphorylation in rat soleus [61,65]. It is notable that after 14-day HS, the increase of AMPK activity (judging by ACC phosphorylation) [62,63] is less pronounced in murine vs rat soleus. 

Now, it is difficult to establish the precise mechanisms that cause an increase in AMPK activity by 14 days of HS. However, some assumptions can be made concerning potential signaling mechanisms leading to this phenomenon. There is evidence that the concentration of interleukin-6 (IL-6) is significantly increased in rodent skeletal muscle after 5- and 14-day HS [66,67] as well as in human skeletal muscle following 60-day bed rest [68]. It is known that IL-6 can increase AMPK activity in rodent skeletal muscle [69], and it is likely that increased concentration of IL-6 during long-term gravitational unloading promotes AMPK hyperphosphorylation. BDNF was also shown to increase AMPK phosphorylation in isolated rat extensor digitorum muscle [30]. Therefore, an increase in BDNF mRNA expression in the spinal cord and soleus muscle of rats after 14 days of HS [70] could be the cause of the increased AMPK Thr172 phosphorylation during this period. 

Thus, HS experiments show complex time-course changes in AMPK activity in the rodent soleus muscle under mechanical unloading. It is important to note that the level of AMPK phosphorylation is significantly reduced during the first day of HS, a period that precedes atrophy development. Additionally, the most likely cause of such a decrease is a shift in the ratio of phosphorylated and dephosphorylated adenine nucleotides (AMP/ATP and ADP/ATP ratios).

## 4. The Role of AMPK in the Regulation of mTOR/p70S6K and Akt/FOXO3/MuRF-1/MAFbx/Atrogin-1 Signaling Pathways under Gravitational Unloading

Anabolic processes in skeletal muscle fibers are regulated by a number of signaling pathways, the most important of which is the mammalian target of rapamycin complex 1 (mTORC1) signaling pathway. mTORC1, through its downstream targets (p70S6K, 4E-BP1), stimulates mRNA translation initiation on a ribosome. Activation of protein synthesis following resistance exercise is associated with the increased level of p70S6K (Thr389) phosphorylation, leading to subsequent phosphorylation of ribosomal protein S6 and initiation of protein synthesis. Some authors reported a decrease in p70S6K (Thr389) phosphorylation in rat soleus after four to five days of HS [71,72]. Other studies showed that even 7–10 days of HS did not affect p70S6K phosphorylation, the level of which decreased only following 14 days of unloading [73,74,75,76].

**Figure 2:**
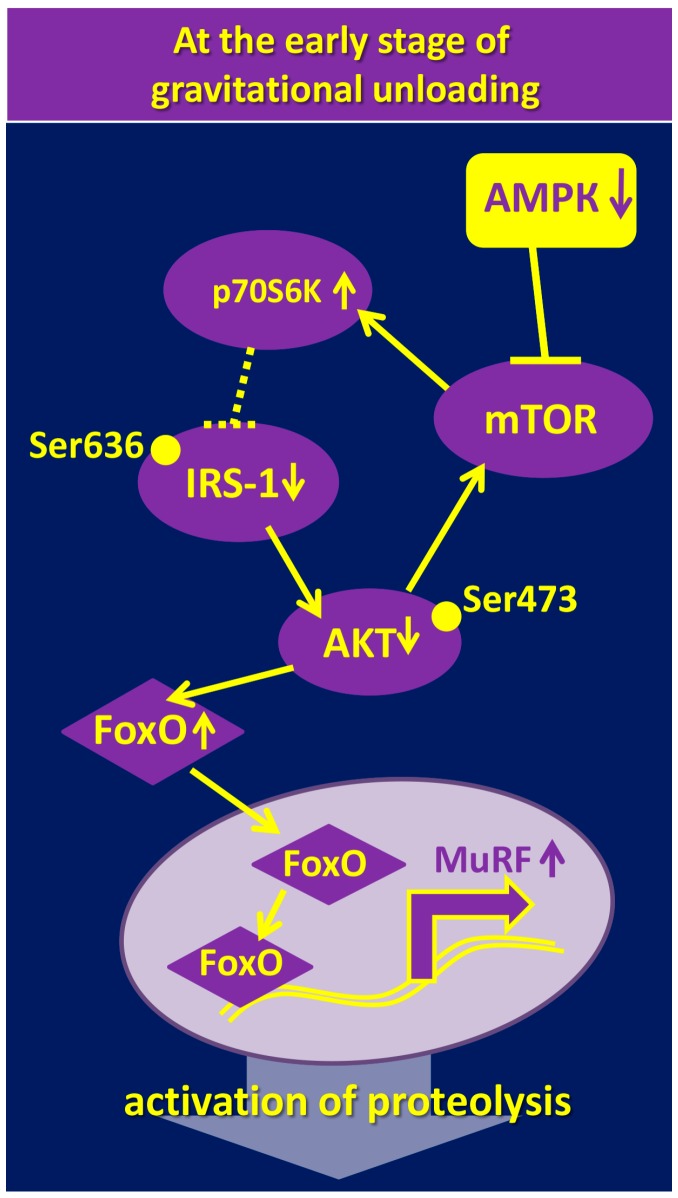
Hypothetical role of AMPK in the activation of signaling pathways regulating the expression of E3-ubiquitin ligases during gravitational unloading.

It is well known that activated AMPK has an inhibitory effect on the anabolic processes via suppression of mTORC1 and its key substrate, p70S6K [41,42,43,77,78,79,80]. Summing up the available data, we can assume that changes in p70S6K and AMPK phosphorylation in rat soleus muscle during the first two weeks of HS show reciprocal relations and complex dynamics. It is important to note that during the early stage of HS (one to three days), an increase in p70S6K Thr389 phosphorylation is accompanied by a decrease in the level of AMPK Thr172 phosphorylation. However, after seven days of HS, there is no difference between these parameters and control values [11]. Two-week HS results in an opposite effect: A decrease in p70S6K phosphorylation is accompanied by an increase in AMPK phosphorylation. These data are consistent with the report by Sugiura and co-authors (2005) that showed no changes in p70S6K phosphorylation after 10 days of HS [74], whereas 14-day HS led to a significant decrease in p70S6K phosphorylation as compared to control levels [71,72,75,76,81]. Interestingly, according to Hilder et al. (2005) and Zhang et al. (2018), the level of AMPK phosphorylation following 14-day unloading is significantly increased [61,65]. The high level of AMPK phosphorylation is accompanied by a decrease in phosphorylation of not only p70S6K, but also Akt and FOXO3, which can lead to an upregulation of muscle-specific E3-ubiquitin ligases, MuRF-1 and MAFbx/atrogin-1 [65]. These results are consistent with earlier studies showing the ability of AMPK to stimulate FOXO3 dephosphorylation and the expression of E3-ubiquitin ligases [44,82]. Time-course changes in the level of p70S6K and AMPK phosphorylation during mechanical unloading suggest that an increase in p70S6K phosphorylation at the first day of HS may be due to the low AMPK activity. This hypothesis has been recently tested by Vilchinskaya and co-authors [83]. Pretreatment of rats with 5-aminoimidazole-4-carboxamide ribonucleotide (AICAR) several days before and during 24-h HS prevented both a decrease in AMPK Th172 phosphorylation as well as an increase in p70S6K Thr389 phosphorylation [83]. This result fully confirmed the hypothesis and once again demonstrated the role of AMPK as a negative regulator of mTORC1-signaling [41,42]. The results obtained by Vilchinskaya et al. (2017) suggest that AMPK dephosphorylation in postural soleus muscle at the initial stage of mechanical unloading is one of the reasons for a paradoxical increase in the level of p70S6K phosphorylation. Increased p70S6K phosphorylation is usually considered to be a consequence of (1) inactivation of endogenous mTORC1 inhibitor tuberous sclerosis complex (TSC1/2) due to AMPK dephosphorylation [41] and (2) accumulation of sphingolipid ceramide [84]. Interestingly, it was previously shown that activation of AMPK with AICAR prevents ceramide accumulation in skeletal muscle fibers [85]. Therefore, it can be assumed that AMPK dephosphorylation can contribute to the accumulation of ceramide in rat soleus under gravitational unloading [86,87,88]. Hsieh and co-authors (2014) observed an interesting effect of p70S6K hyperphosphorylation. The authors showed that activated p70S6K promotes phosphorylation of insulin receptor substrate (IRS1) on Ser636-639, which leads to a reduction in IRS-1 activity and, accordingly, dephosphorylation of downstream protein kinase Akt (Figure 2).

Dephosphorylation of Akt on Ser473, as a rule, causes an increased expression of E3-ubiquitin ligases (MuRF-1 and MAFbx/atrogin-1) [88]. It is well known that even short-term (one to three days) gravitational unloading leads to Akt dephosphorylation and upregulation of E3-ubiquitin ligases [10,66]. According to a number of authors, gravitational unloading results in a significant decrease in IRS-1 content [54,89]. Based on these literature data, it can be assumed that AMPK dephosphorylation during the first day of unloading leads to an increase in p70S6K phosphorylation resulting in the increased E3-ubiquitin ligases’ expression and enhanced proteolysis. Recent experiments with inhibition of p70S6K phosphorylation during the first day of gravitational unloading do not contradict this hypothesis: The use of rapamycin (mTORC1 inhibitor) resulted in a significant decrease in MuRF-1 and MAFbx/atrogin-1 expression [88].

Thus, AMPK dephosphorylation at the initial period of HS leads to an increase in the level of p70S6K phosphorylation, which can contribute to the subsequent upregulation of proteolytic enzymes.

AMPK is known to activate autophagy by ULK phosphorylation (see above). Therefore, one would expect to see a reduction in autophagy markers at the initial period of unloading when AMPK activity is downregulated. However, it has been recently shown that most of autophagy markers, except for ULK, were upregulated in rat soleus following 6- and 12-h HS [64]. The authors of the cited report emphasize that this state of autophagy markers is not consistent with a reduced level of AMPK phosphorylation. They explain this phenomenon by the possible unequal phosphorylation of different AMPK isoforms. It is clear that this issue needs further investigation.

At a later stage of unloading, an increase in AMPK phosphorylation is accompanied by a decrease in p70S6K phosphorylation and activation of the proteolytic signaling system [65].

At the same time, no significant changes in phosphorylation of Akt and p70S6K and the rate of protein synthesis were found in the soleus of transgenic mice overexpressing the dominant-negative mutant of AMPK following 14-day HS [62]. Thus, the lack of AMPK activity in the transgenic mice did not affect anabolic signaling following a relatively long period of unloading (14 days). However, 14-day unloading of these transgenic mice induced a significant increase in the expression of both markers of the ubiquitin-proteasome system and ULK, a marker of autophagy. The differences between dominant-negative AMPK mutants and wild-type mice were significant [62]. There were also significant differences in the severity of atrophic changes. The weight of soleus muscle in dominant-negative AMPK mutants after 14-day HS was significantly reduced, but to a lesser extent than that in wild-type mice (difference of 10–15%) [62,63]. Thus, AMPK can contribute to the development of muscle atrophy during unloading. This contribution to muscle atrophy seems to be carried out via the activation of catabolic processes rather than through the suppression of anabolic regulation.

This fact allows us to suggest that, at later stages of gravitational unloading, AMPK activity can act as a key signaling node of protein homeostasis in skeletal muscle.

## 5. AMPK Is Involved in the Regulation of Myosin Phenotype under Mechanical Unloading

Unloading-induced changes in skeletal muscle myosin phenotype have been observed by a number of researchers. It is well established that there is an increase in the expression of fast-type myosin isoforms and a decrease in the expression of slow-type myosin isoform in skeletal muscles of space-flown rodents as well as astronauts [3,4,90,91]. HS results in a significant increase in the content of fast-twitch (type II) fibers and a significant decrease in the proportion of slow-twitch (type I) fibers in rat soleus muscle [7,92,93,94,95]. In samples of soleus muscle, taken from astronauts after a six-month spaceflight, there was a decrease in the proportion of fibers expressing slow MyHC isoform and an increase in the proportion of fibers expressing fast MyHC isoforms [96]. Mechanisms of molecular regulation of myosin phenotype transformation remain largely unexplored. It is known that AMPK can affect the expression of a number of genes by phosphorylation of class IIA histone deacetylases (HDAC4, HDAC5, HDAC7), which leads to HDACs dissociation from gene promoters and removal from the nucleus, thereby allowing for increased gene expression [48,49,50]. It was earlier shown that at the first day of mechanical unloading, mRNA expression of the slow MyHC isoform in rat soleus is reduced [13]. One of the ways of MyHC expression regulation in muscle fibers is associated with phosphorylation of histone deacetylase 4 (HDAC4) [7,97]. However, it remains unclear what mechanisms are implicated in such a rapid decrease in gene expression. Until recently, there have been no studies on the role of AMPK in the regulation of myosin phenotype in muscle fibers under conditions of gravitational unloading. Can AMPK be involved in the regulation of MyHC gene expression in skeletal muscle fibers? To answer this question, Vilchinskaya et al. (2017) carried out an experiment with AICAR pretreatment of Wistar male rats [98]. There was a significant decrease in MyHC I (β) pre-mRNA expression and a pronounced tendency to a decrease in the mature MyHC I(β) mRNA expression in rat soleus muscle after 24 h of HS. These results are in good agreement with the data previously obtained in skeletal muscles of Sprague-Dawley rats under similar conditions [13]. However, when exposed to AICAR, there were not any significant decreases in the MyHC I(β) pre-mRNA and mature mRNA expression. Since one of the possible mechanisms of AMPK gene expression regulation is linked to histone deacetylase 4 and 5 (HDAC4/HDAC5) phosphorylation status [49,50], it was hypothesized that a significant decrease in AMPK Thr172 phosphorylation after one-day HS would result in HDAC4 and HDAC5 nuclear accumulation [83]. This hypothesis is supported by a previous report that showed a significant deacetylation of histone H3 at the MyHC I (β) gene in rat soleus following seven-day HS [99]. In addition, it has been previously shown that HDAC4 is predominantly localized to the nuclei in fast-twitch fibers in contrast to the sarcoplasm in slow-twitch fibers [100]. Indeed, in our experiment with 24-h HS, HDAC4 accumulation in the nuclear fraction was found; however, in the AICAR-pretreated group, the accumulation of HDAC4 in the nuclei did not occur, which correlates well with data on AMPK phosphorylation and confirms the hypothesis of AMPK-dependent control of nucleocytoplasmic trafficking of HDAC4 [98,101]. Recently, Yoshihara and co-authors have found nuclear accumulation of HDAC4 in rat gastrocnemius muscle following 10 days of ankle joint immobilization. This accumulation (as in the experiment of Vilchinskaya and co-authors) was accompanied by a decrease in the level of AMPK phosphorylation [102]. As for HDAC5, even a slight increase in AMPK activity in the control AICAR-pretreated rat was accompanied by a decrease in HDAC5 content in the nuclear fraction. This phenomenon could be associated with HDAC5 phosphorylation via AMPK. However, 24-h HS resulted in a significant decrease in the nuclear HDAC5 content. Such a reduction in HDAC5 content in the nuclear fraction could be associated with HDAC5 degradation or HDAC5 phosphorylation and subsequent nuclear export. HDAC5 can also be a target for protein kinase D (PKD) [103,104]. Since the AMPK activity is significantly reduced within the first day of unloading (see above), it appears that HDAC5 nuclear export during unloading is not directly linked to AMPK. It has been shown that a decrease in AMPK activity can result in an upregulation of protein kinase D (PKD) phosphorylation [103]. Indeed, one-day HS resulted in a significant increase in PKD Ser916 phosphorylation, however, in the HS+AICAR group, PKD phosphorylation did not differ from the control levels. Interestingly, such a decrease in PKD phosphorylation in the HS+AICAR group vs. the HS group can significantly attenuate the loss of nuclear HDAC5. It is noteworthy that an increase in PKD phosphorylation in unloaded animals did not affect the content of nuclear HDAC4. Obviously, under unloading conditions, HDAC4 does not appear to be a target of PKD. This study was the first to observe the reciprocal relationship between AMPK and PKD in an inactivated skeletal muscle. In addition, gravitational unloading led to an increase in the level of negative AMPK phosphorylation on Ser485/491, which is known to be associated with PKD [35]. However, allosteric activation of AMPK by AICAR reduced the intensity of negative phosphorylation, possibly due to a reduction in PKD phosphorylation.

AMPK is known to stimulate the expression of peroxisome proliferator-activated receptor gamma coactivator 1-alpha (PGC1α), the most important regulator of the expression and activity of signaling proteins. In particular, PGC1α is involved in the control of the expression of the slow isoform of MyHC [105]. A significant decrease in PGC1α expression was found in mouse soleus after four days of hindlimb unloading [106]. However, Vilchinskaya et al. (2017) found no changes in PGC1α mRNA expression after 24-h HS [98]. AMPK activation by AICAR pretreatment also did not induce any changes in PGC1α expression. Obviously, the impact of AMPK on the expression of the slow isoform of MyHC during the first day of unloading is carried out primarily through the trafficking of HDAC4, without the involvement of PGC1α.

**Figure 3:**
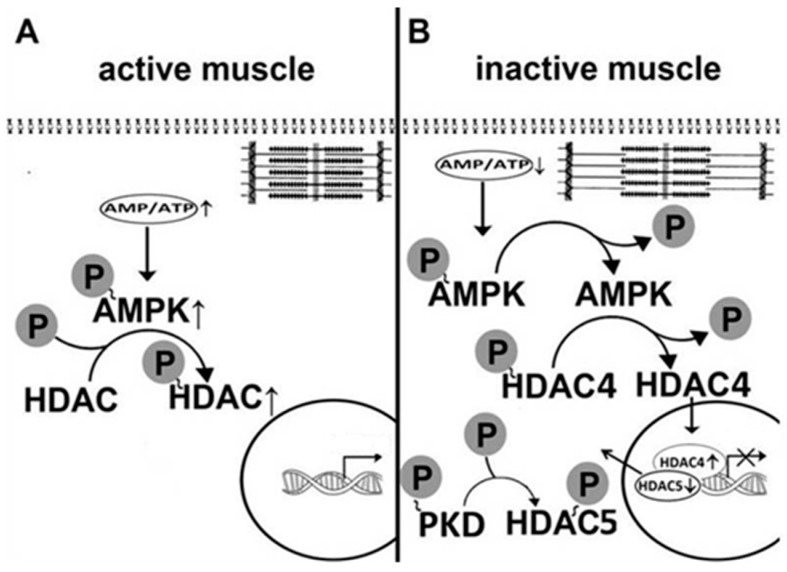
The role of AMPK in class IIa histone deacetylases traffic in rat soleus muscle at the initial stages of gravitational unloading (modified from [98]). (**A**): active muscle; (**B**): inactive muscle. Time-course studies on the MyHCI (β) expression demonstrate that a reduction in MyHCI (β) mRNA expression begins on the first day of unloading and then steadily decreases during, at least, two weeks of hindlimb unloading [13,14]. As shown in dominant-negative AMPK mutants, AMPK has no effect on the expression of slow MyHC after 14-day unloading [63]. It is clear that an experiment with transgenic animals does not allow for tracing the effect of AMPK on the expression of MyHC at the different time-points of unloading. However, the results of this experiment indicate that the decrease in the expression of slow isoform of MyHC after the completion of the initial stage of hindlimb unloading is determined not by AMPK, but by some other mechanisms, for example, via inhibition of the calcineurin/ Nuclear factor of activated T-cells (NFAT) signaling pathway [14,107].

Thus, the results obtained by Vilchinskaya and the co-authors (2017) clearly show that AMPK Thr172 dephosphorylation within the first day of gravitational unloading has a significant effect on the regulation of myosin phenotype in rat postural muscle. In particular, AMPK Thr172 dephosphorylation led to a decrease in MyHC I(β) pre-mRNA and mRNA expression. The study of Vilchinskaya et al. (2017) indicates that HDAC4 is not a target of PKD at the early stages of unloading, and, probably, HDAC4 nuclear import results from a decrease in the AMPK activity (Figure 3). It is possible that an increase in PKD activity can lead to HDAC5 nuclear export.

## 6. AMPK and Disuse-Induced Motor Endplate Remodeling

Reliable neuromuscular transmission is essential for normal bodily functions. Motor endplate is a highly specialized sarcolemma region in which the transmission of activity from motor neurons to striated muscle fiber is realized. Endplate structure, including features of nicotinic acetylcholine receptors’ (nAChRs) localization and distribution, are among the factors crucial for maintenance of highly efficient neuromuscular transmission [108,109].

Ultrastructure of the neuromuscular junction strongly depends on the motor activity, and exhibits high morphological and functional plasticity. Different modes of increased activity are manifested in the morphological remodeling, such as the expansion of the neuromuscular junction size. Reduced patterns of neuromuscular activity also trigger endplate remodeling. Alterations in neuromuscular junction stability and integrity progressively increase with age [110,111,112,113,114] and myodystrophy [115,116,117], in animal models of muscle injures [118,119], after denervation [120] and prolonged (four weeks) HS [121]. Although the molecular mechanisms underlying the structural and functional endplate plasticity are intensively studied, they are not completely clear [113,115,116,122,123,124,125].

Currently, many facts point to the involvement of AMPK in neuromuscular junction remodeling. AMPK is linked to a variety of cellular processes and is also considered a crucial activator of autophagy and its downstream target, ULK1 [126]. Autophagy is involved in neuromuscular junction preservation during aging, and impairments in autophagy exacerbate synaptic structure degeneration [111]. AMPK and AMPK-activated autophagy are among the most important factors that maintain neuromuscular junction stability [111,127,128]. Pharmacological activation of AMPK by AICAR administration improves integrity of neuromuscular junctions and prevents skeletal muscle pathology in a mouse model of severe spinal muscular atrophy [128]. Additionally, AICAR treatment has been shown to stimulate autophagy and ameliorate muscular dystrophy in the mdx mice, a model of Duchenne muscular dystrophy, suggesting AMPK as a powerful therapeutic target [129,130]. Also, AMPK may play a role in activating PGC-1α, a key regulatory protein in skeletal muscle adaptation to physical activity. PGC-1α has been reported to play a major role in maintaining neuromuscular junction integrity [122,123].

AMPK also plays a beneficial role in the regulation of skeletal muscle cholesterol synthesis as well as sarcolemma cholesterol levels [131,132]. Direct molecular interaction between membrane cholesterol and the nAChRs has been shown [133]. Moreover, cholesterol and lipid rafts contribute to the orchestration of nAChRs clustering at the endplate region [134,135]. In addition, AMPK can affect the Na,K-ATPase activity [38,39]. The targeting and activity of the Na,K-ATPase are also influenced by the cholesterol environment [136,137,138,139], and reciprocal interactions between cholesterol and the α2 Na,K-ATPase isozyme has been suggested [22,140]. Notably, both the nAChRs and the α2 Na,K-ATPase isozyme are enriched at the endplate region, co-localized, and co-immunoprecipitated, suggesting that these proteins exist as a functional multimolecular complex to regulate electrogenesis and to maintain the effectiveness of neuromuscular transmission [141,142,143].

The loss of the α2 Na,K-ATPase isozyme electrogenic activity accompanied by disturbances in lipid rafts and endplate structure stability were observed in rat soleus muscle even after 6–12 h of HS [21,22,64]. Such acute disuse decreased the endplate area and increased the density of the nAChRs distribution. These changes were accompanied by decreased phosphorylation of AMPK and its substrate, ACC, and increased autophagy [64]. Autophagy is known to be involved in the nAChRs turnover regulation [127] and has a major impact on neuromuscular synaptic function [111]. So, an increase in autophagy after acute HS can reflect an adaptive response to compensate for the endplate area loss through increasing the density of the nAChRs distribution [64].

Notably, pretreatment of the rats with AMPK activator, AICAR, followed by HS, stabilized the resting membrane potential, endplates area, and the nAChRs distribution density, indicating that AMPK activation can prevent disuse-induced endplate structural and functional reorganization (unpublished observation).

In summary, these novel findings indicate that endplate functional and structural characteristics rapidly (within hours) respond to skeletal muscle disuse. Decreased phosphorylation of AMPK accompanied by increased autophagy is the earliest disuse-induced remodeling event preceding the overt skeletal muscle atrophy.

## 7. Conclusions

AMPK demonstrates multidirectional changes in a mammalian soleus muscle during gravitational unloading: A deep decrease in the level of phosphorylation and kinase activity at the early stages of the process and a significant increase in activity at the later stages. Time-course changes in AMPK activity under unloading are nonlinear and require a more detailed analysis at each stage of the process. The experiments discussed in this review show that changes in AMPK activity under unloading conditions can have a significant impact on the key signaling pathways and molecular structures in skeletal muscle fiber. As a result of reduction in AMPK activity at the initial stage of unloading, paradoxical hyperphosphorylation of p70S6K occurs, which, according to in vitro experiments, can lead to the activation of proteolytic processes in muscle fiber [83,87]. A decrease in the level of phosphorylation and kinase activity of AMPK at the early stages of unloading also affects the change in nucleocytoplasmic traffic of class IIA HDACs, resulting in a decrease in the expression of the myh7 gene (slow isoform of MyHC) and, possibly, a number of other genes controlling energy metabolism. At the later time-points of unloading, an increase in the level of AMPK phosphorylation/activity is accompanied by a decrease in the activity of p70S6K [76], FOXO3 dephosphorylation, and an increase in the expression of the key enzymes of the ubiquitin-proteasome pathway [65], which, obviously, should lead to decreased muscle protein synthesis and enhanced proteolysis. Unfortunately, to date, the precise physiological mechanisms, both intramuscular and systemic, underlying such a complex and nonlinear nature of AMPK activity during gravitational unloading are not known. It is possible that glycogen accumulation [27] or a change in the ratio of dephosphorylated and phosphorylated high-energy phosphates could lead to a deep AMPK dephosphorylation. The cause of a gradual increase in AMPK phosphorylation in rat soleus during the first week of HS remains unclear. It is possible that this increase is due to a gradual increase in the electromyographic activity of the postural muscle [15]. It is difficult to explain a significant increase in AMPK phosphorylation in rat soleus by the end of the second week of HS. Possibly, such systemic factors as BDNF and/or interleukin-6 could play a role in these processes. All these questions have yet to be answered.

Thus, recent studies have revealed the key role of AMPK in the processes of deep remodeling of signaling pathways, leading to changes in metabolism, structure, and function of postural muscle fibers under unloading conditions. Further studies are needed to elucidate new signaling mechanisms that trigger, determine, and limit atrophy development and intrinsic muscle stiffness, as well as myosin phenotype changes, in a mammalian postural muscle under mechanical unloading.

## Abbreviations

AMPK	5′ adenosine monophosphate -activated protein kinase
AICAR	5-aminoimidazole-4-carboxamide ribonucleotide
BGPA	β-Guanidinopropionic acid
HS	hindlimb suspension
mTOR	mammalian/mechanistic target of rapamycin
p70S6K	ribosomal protein S6 kinase beta-1
CaMKKβ	Ca(2+)/calmodulin-dependent protein kinase kinase β
LKB1	liver kinase B1
BDNF	brain-derived neurotrophic factor
MuRF-1	Muscle RING-finger protein-1
FOXO	forkhead box proteins
IRS-1	insulin receptor substrate 1
MyHC	myosin heavy chain isoforms
nAChRs	nicotinic acetylcholine receptors
ULK-1	Unc-51-like kinase
PGC1α	peroxisome proliferator-activated receptor gamma coactivator 1-alpha
